# Anesthetic Management for Resection of a Cerebellar Hemangioblastoma Leading to Brainstem Compression in a Patient With Von Hippel-Lindau Disease

**DOI:** 10.7759/cureus.20608

**Published:** 2021-12-22

**Authors:** Christiano Dos Santos e Santos, Guilherme dos S. e Santos, Cristiane Araujo Tuma Santos

**Affiliations:** 1 Anesthesiology, University of Mississippi Medical Center, Jackson, USA; 2 Anesthesiology, Miguel Couto Hospital, Rio de Janeiro, BRA; 3 Radiology, University of Mississippi Medical Center, Jackson, USA

**Keywords:** brainstem compression, von hippel-lindau syndrome, neuromonitoring, cerebellar hemangioblastoma, anesthetic management

## Abstract

Von Hippel-Lindau (VHL) disease is a complex genetic syndrome characterized by multisystemic vascular neoplastic disorder. The affected population tends to develop tumors mainly involving the central nervous system, adrenal glands, pancreas, and kidneys. We describe anesthetic management for the resection of a cerebellar mass compressing the brainstem in a recently diagnosed 25-year-old female patient with a history of von Hippel-Lindau (VHL) syndrome. An uneventful occipital craniectomy for cerebellar tumor resection was performed under total intravenous anesthesia, without complications. The patient was discharged home on postoperative day five. This case depicts a situation in which a brainstem compressing lesion needs to be addressed urgently, and the pharmacological neuroprotective technique utilized for this procedure.

## Introduction

The multiorgan, autosomal dominant, hereditary neoplastic syndrome named von Hippel-Lindau [1] was initially described in 1925 by the Swedish physician Arvid Vilhelm Lindau in his studies on the pathogenesis of cerebellar cysts and their relationship with retinal angiomatosis [2]. Three years later, the term angioblastoma was introduced into the literature by Drs. Cushing and Bailey [3]. The disorder was initially named Lindau's disease and acknowledged in the United States following Dr. Cushing's publication of "Hemangiomas of cerebellum and retina (Lindau's disease): with the report of a case [4]."

The syndrome occurs due to a germline mutation observed in the tumor suppressor gene VHL located on the short arm of chromosome 3 (3p25-26) [5-6]. It is not considered rare since it presents an incidence of approximately one in 36,000 live births [7] with observed penetrance by 65 years of age of over 90% [8]. The above-described mutation predisposes the affected population to develop lesions in the central nervous system (CNS) and visceral organs [1]. The most commonly observed lesions in the CNS are retinal hemangioblastomas, endolymphatic sac tumors, and craniospinal hemangioblastomas (cerebellum, brainstem, spinal cord, lumbosacral nerve roots, and supratentorial) while the visceral lesions usually observed are renal cell carcinomas and cysts, pheochromocytomas, pancreatic tumor or cyst, and ependymal cystadenoma [9-10].

VHL inherited (familial) corresponds to 80% of the affected patients while 20% of VHL cases are associated with new and sporadic mutations [1]. Symptoms present in the second decade of life are considered early manifestations of the syndrome, with approximately 50% of this population expressing clinical manifestations at the initial presentation [1]. The most typical initial symptom of VHL is related to cerebellar hemangioblastomas [11]. Patients with VHL syndrome present an average life expectancy of 59.4 years for men and 48.4 years for women [12]. Complications associated with renal cell carcinomas and hemangioblastomas of the central nervous system are the principal causes of death [8-13].

The diagnosis of VHL syndrome is generally established on clinical elements such as a positive family history of VHL disease and the existence of at least one VHL-associated tumor [1]. The disease is classified into types and subtypes accordingly to distinguishing genotype-phenotype features observed in the affected patients [1]. The initial classification into types 1 and 2 are entrenched on the risk (increased or decreased) of developing a pheochromocytoma [14]. Type 1 has a decreased risk for pheochromocytoma development while retinal and CNS hemangioblastomas, renal cell carcinomas, pancreatic cysts, and neuroendocrine tumors are other common clinical findings [1-14]. Furthermore, type 2 presents an increased risk of pheochromocytoma development, and its subtypes are associated with the risk of renal cell carcinoma development [1-14]. The subtype 2A presents a low risk of renal cell carcinoma, 2B shows a high risk of renal cell carcinomas, while subtype 2C only presents pheochromocytoma without any other tumor [1-14]. Once the condition is diagnosed, multidisciplinary care reinforcing screening guidelines will lead to early detection of the disorder's clinical features and consequent reduction of morbidity and mortality rates [15].

The most commonly observed tumor in this syndrome is the hemangioblastoma of the CNS, present in 60-80% of all affected individuals [16]. Hemangioblasts are the probable origin of the CNS hemangioblastomas observed in this disease [17]. The preferred imaging study for CNS hemangioblastomas detection is magnetic resonance imaging following intravenous contrast administration [18]. Even though histologically benign, significant morbidity can occur due to compression and perilesional edema of non-silent areas of the CNS [5]. Although hemangioblastomas can appear in any portion of the CNS, they are most frequently observed in the spinal cord, cerebellum, and brainstem with tumor size and location being accountable for the clinical features noticed in each patient [5]. The hemangioblastoma behavior is unpredictable, with rapid expansion alternating with tumoral hibernation, characteristically described as a saltatory growth pattern [19]. For this reason, the surgical approach, which is customarily considered curative, is delayed until the patients become symptomatic [20].

## Case presentation

**Figure 1:**
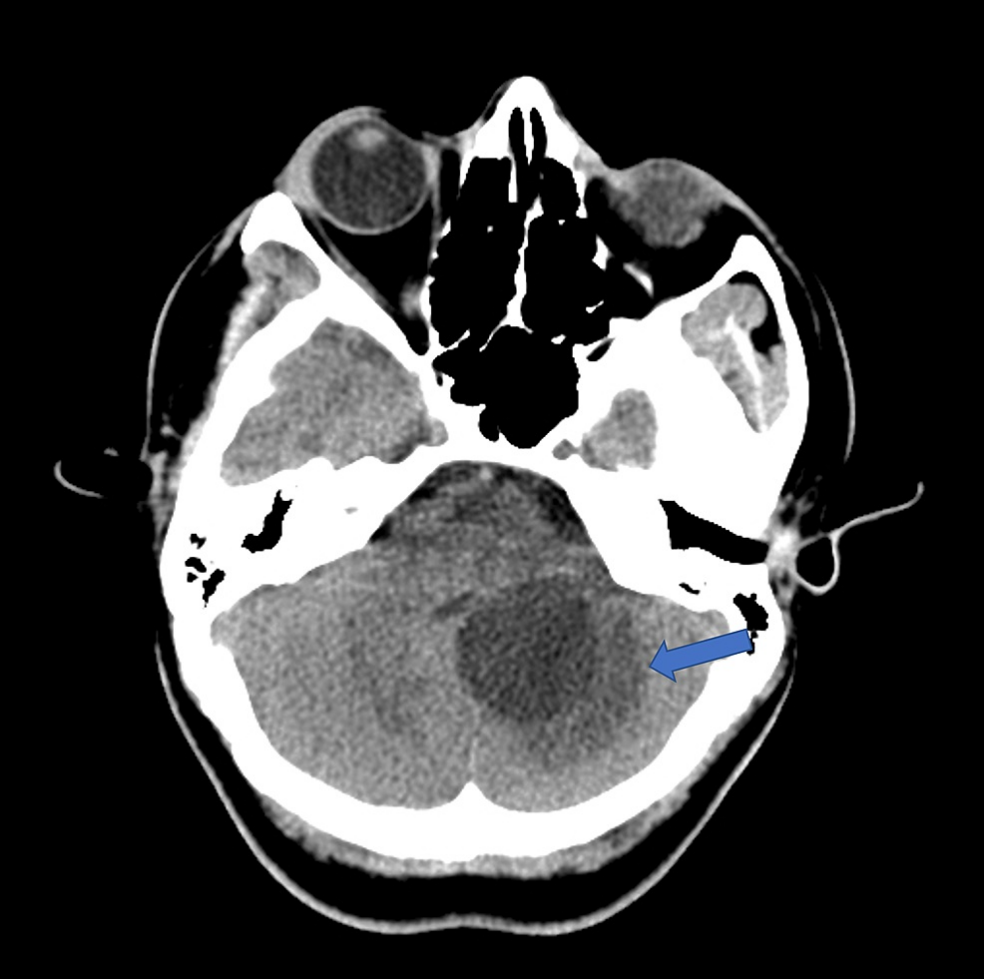
Axial CT without contrast Three cm cystic lesion in the posterior fossa, centered in the left medial cerebellar hemisphere with mass effect on the fourth ventricle and surrounding edema (blue arrow)

**Figure 2:**
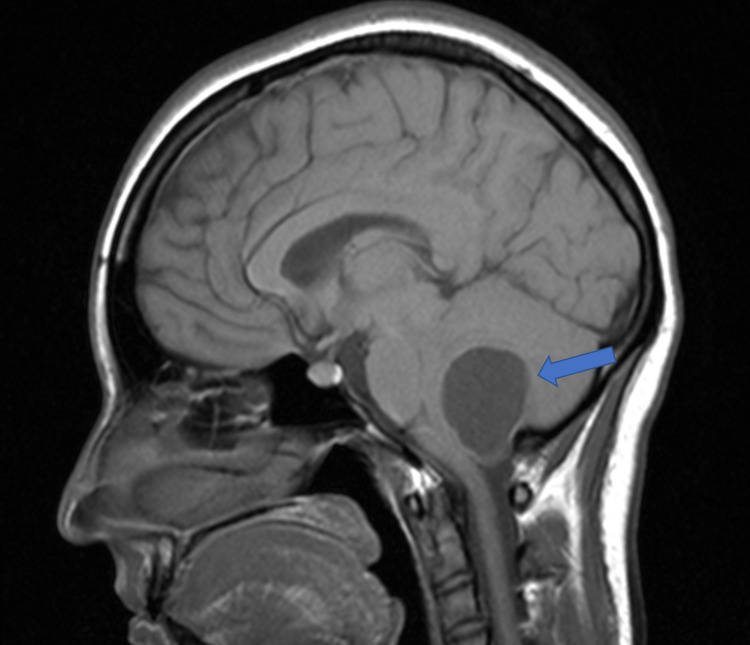
Sagittal T1 MRI without contrast Cystic lesion in the cerebellar hemisphere with associated mass effect and compression of the fourth ventricle and dorsal brainstem (blue arrow)

**Figure 3:**
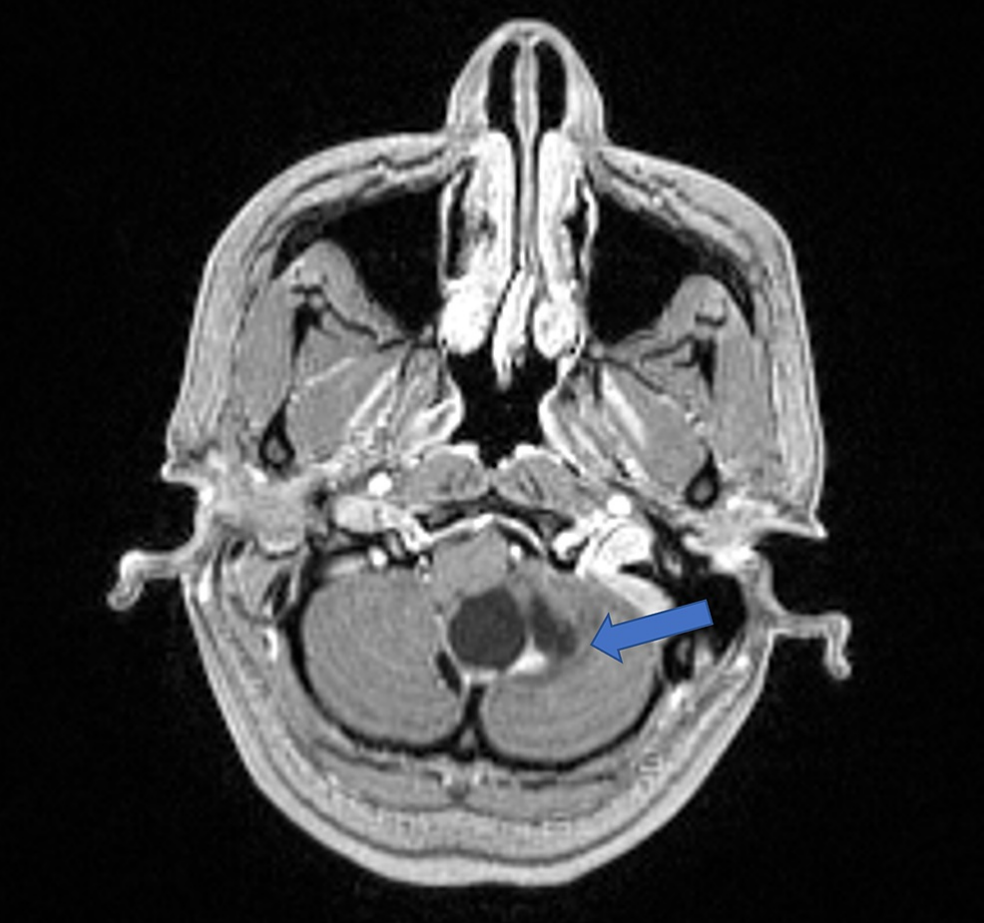
Axial T1 MRI with contrast Six mm solid enhancing component in the inferior aspect of the lesion (blue arrow)

This article describes successful anesthetic management of a patient with a voluminous posterior fossa cystic lesion located in the cerebellum. The patient was a 25-year-old female (69 kg; 160 cm; BMI 26.95), with a history of von Hippel-Lindau (VHL) syndrome that was recently diagnosed (six months earlier), who presented to the emergency department (ED) complaining of nausea, vomiting, blurry vision, and unsteadiness in her feet, leading to gait instability for the last five days. Computed tomography of the head revealed a cerebellar cystic tumor (Figure 1) and a brain MRI following admission showed a 6 mm tumor with solid enhancing component associated mass effect and compression of the fourth ventricle and dorsal brainstem (Figures 2-3). The patient's father had VHL disease and was deceased 12 months before this visit to the ED due to complications of renal cancer. She denied any previous surgeries and hospitalizations. The patient was admitted to the neurological intensive care unit (NSICU) with multispecialty consideration led by the neurosurgical department. Ophthalmology and endocrinology were consulted to rule out other common manifestations of VHL disease such as either retinal hemangioblastoma or pheochromocytoma. Extensive laboratory workup and imaging study were executed. No other concomitant lesion was observed, and an occipital craniectomy for a cerebellar cystic mass resection was scheduled. Due to the vascular nature of the tumor, blood transfusion consent was acquired formerly to type and screen and cross-match for two units of packed red blood cells (pRBC), and two units of fresh frozen plasma (FFP) was requested. After informed consent was obtained, the patient was transferred to the main operating room area.

Preoxygenation was initiated following placement of the standard American Society of Anesthesiologists monitorization. The pre-induction vital signs were within normal limits. General endotracheal anesthesia was induced with the administration of lidocaine 1% (1 mg/kg), fentanyl (1 µg/kg), propofol (3 mg/kg), and succinylcholine (1 mg/kg). A secure airway was established with direct laryngoscopy showing Cormack-Lehane grade I view [21], followed by a bite block placement. The surgical-anesthetic plan included somatosensory-evoked potentials (SSEP) and motor-evoked potentials (MEP), conducted by the neurophysiology team. The neuromonitoring plan determined the neuromuscular block agent (depolarizing) and the anesthetic maintenance (total intravenous anesthesia). After endotracheal intubation, a radial arterial line was placed, followed by blood sample analysis (baseline arterial blood gas and thromboelastography). The anesthetic maintenance was achieved with propofol (50-80 µg/kg/min) and remifentanil (0.08-0.1 µg/kg/min) infusions. Head holder (Mayfield) placement occurred after a three-point injection of ropivacaine 0.5% (total of 15 milliliters). The patient was carefully placed in a prone position, and an uneventful occipital craniectomy for a cerebellar mass resection was successfully performed. By neurosurgeon request, intravenous mannitol was administered (0.5 mg/kg) in 10 minutes. The patient received 1.5 L of normal saline throughout the case. Urine output was 800 ml. The estimated blood loss was 500 ml. Both neuromonitoring modalities were unremarkable throughout the case. T1he patient was extubated at the end of the procedure, and pain control was accomplished using intravenous hydromorphone 0.5 mg. Dexmedetomidine 1 µg/kg (10 minutes infusion) was administered to prevent remifentanil-induced hyperalgesia. Subsequently, the patient was transferred to NSICU where she stayed for the next 24 hours. Soon after, she was moved to the floor and discharged home on postoperative day five.

## Discussion

The anesthetic management of patients with elevated intracranial pressure (ICP) is always challenging. It is well-known that cerebral perfusion pressure (CPP) is the mean arterial pressure (MAP) minus ICP (CPP = MAP - ICP) [22]. The scenario described here is even more complicated because of the elevated pressure localized in the posterior fossa. The mass effect on the fourth ventricle and brainstem compression are important radiological points to consider. They reassure the anesthesiologist in charge of the case that the anesthetic plan must contemplate a pharmacological approach capable of promoting the reduction of the cerebral blood flow with consequent decrease of ICP and elevation of CPP. Another critical piece of information provided by radiology is the presence of perilesional edema. It suggests that the intravenous administration of a potent osmotic diuretic such as mannitol 20% (0.5-1 g/kg) would be extremely valuable in alleviating the infratentorial pressure. It also would promote optimization of the brainstem perfusion due to reducing the mass effect and compression [23].

The neuroprotective properties of propofol administration through continuous intravenous infusion during surgery promote the activation of the type A gamma-aminobutyric acid (GABA) receptor with the consequent opening of chloride channels and neuronal hyperpolarization and protecting the CNS against oxidative stress [24]. This decline of metabolic demand is crucially related to diminishing the cerebral blood flow (CBF) without neuronal or glial hypoxic-ischemic consequences. Since this is a short-acting anesthetic agent with antiemetic properties, extubation at the case's end is expected. Additionally, the risk of propofol infusion syndrome is mitigated once the drug is administrated intraoperatively only [25].

Remifentanil is an ultra-short-acting opioid responsible for promoting rapid anesthetic emergence compared with other opioids, such as fentanyl and sufentanil, promoting immediate neurologic physical exam after extubation [26]. Remifentanil is quickly metabolized by non-specific esterases located in the blood and tissues, and it is unable to impair the CNS responsiveness to carbon dioxide variations [26]. The association between remifentanil and propofol does not affect the cerebral blood flow velocity and preserves CBF autoregulation, which offers outstanding neuroprotection even in adverse situations. A disadvantage of remifentanil administration is the increased risk of postoperative opioid-induced hyperalgesia. The literature has effectively described its prevention by the intravenous administration of dexmedetomidine (1 µg/kg in 10 minutes infusion) [27].

The medical literature has vastly suggested the benefits of total intravenous anesthesia (TIVA) improving CPP due to the reduction of CBF and, consequently, the reduction of ICP compared to the administration of volatiles during neurosurgical procedures [28].

Neurophysiology monitoring during surgery is paramount when essential parts of the CNS are at risk of intraoperative injury. It can be affected by the depth of anesthesia and a closed-loop communication between anesthesiologist and neurophysiology is crucial to avoid this undesirable intraoperative event. The correct identification of neural structures, such as cranial nerves and ascending and descending tracts, related to sensibility and motor function is directly related to decreased morbidity [29].

## Conclusions

Although VHL syndrome is linked to benign tumors in the CNS, these hemangioblastomas can bear a potential neurological devastating situation due to their location and unpredictable growth. Since the surgical approach is reserved for the symptomatic population, the anesthesiologist will always face a patient with impaired neurophysiology requiring extensive neuropharmacological understanding. Neuroprotective techniques will enormously benefit the patient by decreasing morbidity and mortality rates. Multispecialty consideration is encouraged due to the complexity of the disease and its life-threatening features. We strongly suggest that the anesthetic management of these CNS lesions would benefit from TIVA compared to balanced general anesthesia. The benefit of propofol and remifentanil association outweigh these drugs' risks and side effects.
